# Perceived stress linking psychosocial factors and depressive symptoms in low-income mothers

**DOI:** 10.1186/s12889-020-10118-4

**Published:** 2021-01-06

**Authors:** Mei-Wei Chang, Roger Brown, Duane T. Wegener

**Affiliations:** 1 College of Nursing, The Ohio State University, 1585 Neil Avenue, Columbus, OH 43210 USA; 2 School of Nursing, University of Wisconsin-Madison, 701 Highland Ave, Madison, WI 53705 USA; 3 Department of Psychology, The Ohio State University, 1835 Neil Avenue, Columbus, OH 43210 USA

**Keywords:** Stress, Autonomous motivation, Coping self-efficacy, Emotional coping, Social support, Depression

## Abstract

**Background:**

Little is known about associations between perceived stress, psychosocial factors (social support, emotional coping, coping self-efficacy, and autonomous motivation), and depressive symptoms in low-income overweight or obese mothers of young children. Using baseline data of a lifestyle intervention study, this secondary analysis investigates whether perceived stress might mediate the associations between the psychosocial factors and depressive symptoms.

**Methods:**

Convenience sampling was applied. Low-income overweight or obese mothers of young children were recruited from the Special Supplemental Nutrition Program for Women, Infants, and Children in Michigan, US. Survey data were collected through phone interviews. Participants (*N* = 740) responded to valid surveys measuring perceived stress, social support, emotional coping, coping self-efficacy, autonomous motivation, and depressive symptoms. Composite indicator structural equation modeling was performed to test for potential mediation.

**Results:**

When investigating the potential role of perceived stress as a mediator, the indirect effects of social support (b = − 2.10, *p* < 0.01), emotion coping (b = − 3.81, *p* < 0.05), and coping self-efficacy (b = − 7.53, p < 0.01) on depressive symptoms through perceived stress were significant, but the indirect effect of autonomous motivation was not.

**Conclusion:**

Future intervention studies aiming to alleviate depressive symptoms in low-income overweight or obese mothers of young children might consider including practical strategies to promote social support, emotional coping, and coping self-efficacy to reduce perceived stress, which might potentially decrease depressive symptoms.

**Trial registration:**

Clinical Trials NCT01839708; registered February 28, 2013.

## Background

Depression substantially disrupts the daily home or work life of low-income mothers of young children [1]. Such disruption of daily life increases risk for poor interactions between mothers and their young children [1], which subsequently influences children’s well-being. For example, evidence has shown that maternal depression is associated with children’s emotional and behavioral problems [2], poor cognitive skills, [3] and risk for depression [4]. Depression in adults is strongly associated with obesity [5], which is also associated with numerous chronic conditions, such as type 2 diabetes and cardiovascular disease [6]. Approximately 58% of low-income overweight or obese mothers of young children reported clinically significant levels of depressive symptoms [7]. When depression and obesity co-occur, they can be increasingly difficult to treat [8, 9], which underscores the critical and urgent needs of alleviating depressive symptoms in low-income overweight or obese mothers of young children, our priority population.

A potential contributor to depressive symptoms in low-income mothers of young children is perceived stress [10–14]. High levels of perceived stress are highly prevalent in the priority population [15]. Fortunately, depressive symptoms can be reduced [16] for low-income mothers of young children through stress management [17]. One step towards ultimately developing an effective theory-based stress management intervention aimed at alleviating depressive symptoms in the priority population is to test potentially useful theories. Testing such theories can advance science and allows researchers to identify potential underlying mechanisms of change (for example, mediators of intervention effects) [18]. This process subsequently enables researchers to modify the intervention to make it more effective. An important factor affecting behaver change is motivation [19] and the Self-Determination Theory gives motivation a primary role in behavioral change [19]. Therefore, we chose this theory to guide the conceptual framework of the current study. This theory includes 3 key constructs: autonomous motivation, relatedness with others, and competence [20]. The Self-Determination Theory [20] postulates that autonomous motivation (defined as personal interests or values to adopt or maintain a behavior), relatedness with others (tested here in terms of social support), coping (tested here in terms of emotional coping), and competence (tested here in terms of self-efficacy) are all associated with perceived stress. Previous intervention studies aim to reduce depression symptoms via promotion of stress management have not addressed social support, emotional coping, and coping self-efficacy.

Autonomous motivation has been strongly recommended to be included in interventions aimed at reducing perceived stress [21]. Yet, only one study of college students has investigated the association between autonomous motivation and perceived stress [22]. Prior studies of mothers of young children have investigated the association between social support and various types of stress such as adverse life event stress [12], parenting stress [23], and economic stress [24]. However, the association between social support and overall perceived stress remains unknown. Emotional coping refers to strategies used to lessen emotional distress related to stressful situations. Emotional coping might represent the most effective means of reducing perceived stress. Also, emotional coping might be of particular relevance here because, unlikely problem-focused coping, it can be used even when the person cannot control the situations they face [25], but no previous research has examined the association between emotional coping and perceived stress in our priority population. Self-efficacy refers to one’s confidence in performing a task or behavior [26]. Only one study investigated the association between self-efficacy (measured as parental self-efficacy) and stress (measured as parenting stress) in mothers of young children [11]. The association between coping self-efficacy (confidence in effectively coping with stressful situations) and overall perceived stress remains under-investigated.

In terms of the associations between the four psychosocial factors and depression, no studies have investigated the association between autonomous motivation and depressive symptoms in mothers of young children. Only a few studies have investigated the association between social support and depressive symptoms [12, 27], the association between emotional coping and depressive symptoms [28], and the association between self-efficacy -- measured as parental self-efficacy [29, 30] or general self-efficacy [31, 32] -- and depressive symptoms in mothers of young children.

To date, limited research has tested the Self-Determination Theory constructs, and no study has used the theory as a basis for linking psychosocial factors to depression through stress in the priority population. Therefore, in the following sections, we describe participants, measures, statistical analyses, and results used to investigate the hypothesis that stress plausibly mediates the association between social support, emotional coping, coping self-efficacy, autonomous motivation, and depressive symptoms in low-income overweight or obese mothers of young children. We conclude by noting conceptual and applied implications of the research.

## Methods

### Design, participants, and setting

This secondary analysis utilized baseline data (*N* = 740) from a community-based lifestyle behavior intervention study of low-income overweight or obese mothers of young children. Detailed descriptions of recruitment, study criteria, and procedures have been described elsewhere [33, 34]. Briefly, participants were recruited from the Special Supplemental Nutrition Program for Women, Infants, and Children (WIC) in Michigan, U.S. To be eligible for enrollment in the study, women had to be non-Hispanic Black or White, have a body mass index (BMI) between 25.0 and 39.9 kg/m^2^ (calculated using measured height and weight), be between 18 and 39 years old, be not pregnant, and be between 6 weeks and 4.5 years postpartum. All eligible participants provided a written informed consent prior to enrollment. Michigan State University Institutional Review Board approved the Study procedures.

### Measures

Study-trained phone interviewers collected survey data related to the study variables via phone and entered data simultaneously to a computer data collection system, which was programed not to allow any missing data. Of surveys used for the current study, three of them (social support, emotional coping, and coping self-efficacy, described below) were developed and tested in the priority population. Briefly, we initially generated the survey items based on our focus group discussions [35]. Next, we conducted individual face-to-face cognitive interviews, which tested participants’ understanding of the survey questions, to establish face validity. After that, we administered the survey to women who did not participate in the focus group discussion or cognitive interviewing testing studies. Finally, we performed exploratory and confirmatory factor analyses to establish validity and reliability [36].

#### Independent variable. Social support for stress management (hereafter, social support)

The social support survey has possessed construct and convergent validity and reliability (Cronbach’s alpha (α) = 0.87) [36]. Participants were asked to respond to 6 items using a 1 (rarely or never) to 4 (usually or always) scale to rate the frequency of getting support when needed. For example, “you can rely on family members, friends or others when you need to ‘talk about problems at work or home.” The scores from the 6 items were averaged, with higher scores reflecting more social support (present study: M = 2.56, SD = 0.78, α = 0.88).

#### Independent variable. Emotional coping for stressful situations (hereafter, emotional coping)

The emotional coping survey has evidenced construct validity and reliability (α = 0.80–0.91) [36]. Participants were asked to respond to 5 items using a 1 (rarely or never) to 4 (usually or always) scale to assess strategies used to reduce negative emotions during stressful situations. For example, “how often do you deal with or prevent stress by ‘doing exercise, for example, going for a walk.’” The scores from the 5 items were averaged, with higher scores reflecting more positive emotional coping (present study: M = 2.89, SD = 0.53 α = 0.60).

#### Independent variable. Coping self-efficacy for stress management (hereafter, coping self-efficacy)

The coping self-efficacy survey has shown construct validity and reliability (α = 0.92) [36]. Participants were asked to respond to 10 items using a 1 (not at all confident) to 4 (very confident) scale to assess confidence in ability to relax during stressful situations. For example, “you can relax, even when ‘you are stressed.’” The scores from the 10 items were averaged, with higher scores reflecting higher coping self-efficacy (present study: M = 2.30, SD = 0.58 α = 0.86).

#### Independent variable. Autonomous motivation for stress management (hereafter, autonomous motivation)

Autonomous motivation was assessed using the Treatment Self-Regulation Questionnaire. The survey has evidenced construct validity and reliability (α = 0.92) [37]. Participants were asked to respond to 6 items using a 1 (not at all true) to 7 (very true) scale to assess their personal value or interest in engaging activities to manage stressful situations. For example, “the reason I practice better ways to deal with stress is ‘because I want to take responsibility for my own health.’” The scores from the 6 items were averaged, with higher scores reflecting higher autonomous motivation (present study: M = 5.43, SD = 1.33, α = 0.90).

#### Mediator. Perceived stress

Perceived stress was assessed using the revised Perceived Stress Scale, the most widely used psychological survey for measuring the perception of stress. The survey has shown predictive validity and good reliability (α = 0.72) [38]. Participants were asked to respond to 9 items using a 1 (rarely or never) to 4 (usually or always) scale to rate the degree to which situations in one’s life have been appraised as stressful in the last month. For example, “how often have you felt that ‘you were unable to control the important things in your life.” The scores from the 9 items were averaged, with higher scores reflecting higher levels of perceived stress (present study: M = 2.53, SD = 0.48, α = 0.76).

#### Dependent variable. Depressive symptoms

Depressive symptoms were assessed using the Center for Epidemiologic Studies Depression Scale (CES-D), one of the most widely used surveys to measure depressive symptoms. This survey has concurrent validity and reliability (α = 0.85–0.90) [39]. Participants were asked to respond to 20 items using a 0 (rarely or none of the time or less than 1 day) to 3 (most or all of the time or 5–7 days) scale to assesses self-reported symptoms associated with depression experience in the last week. For example, “during the past week, ‘I felt that I could not shake off the blues even with help from my family or friends.’” The scores from the 20 items were summed, with higher scores reflecting more depressive symptoms (present study: M = 19.98, SD = 10.34, α = 0.90).

Covariates. Demographic data included age, postpartum period, race, smoking, education, and employment status. These data were collected through a pencil-and-paper survey while women waited for their WIC appointments.

### Statistical analysis

**Fig. 1:**
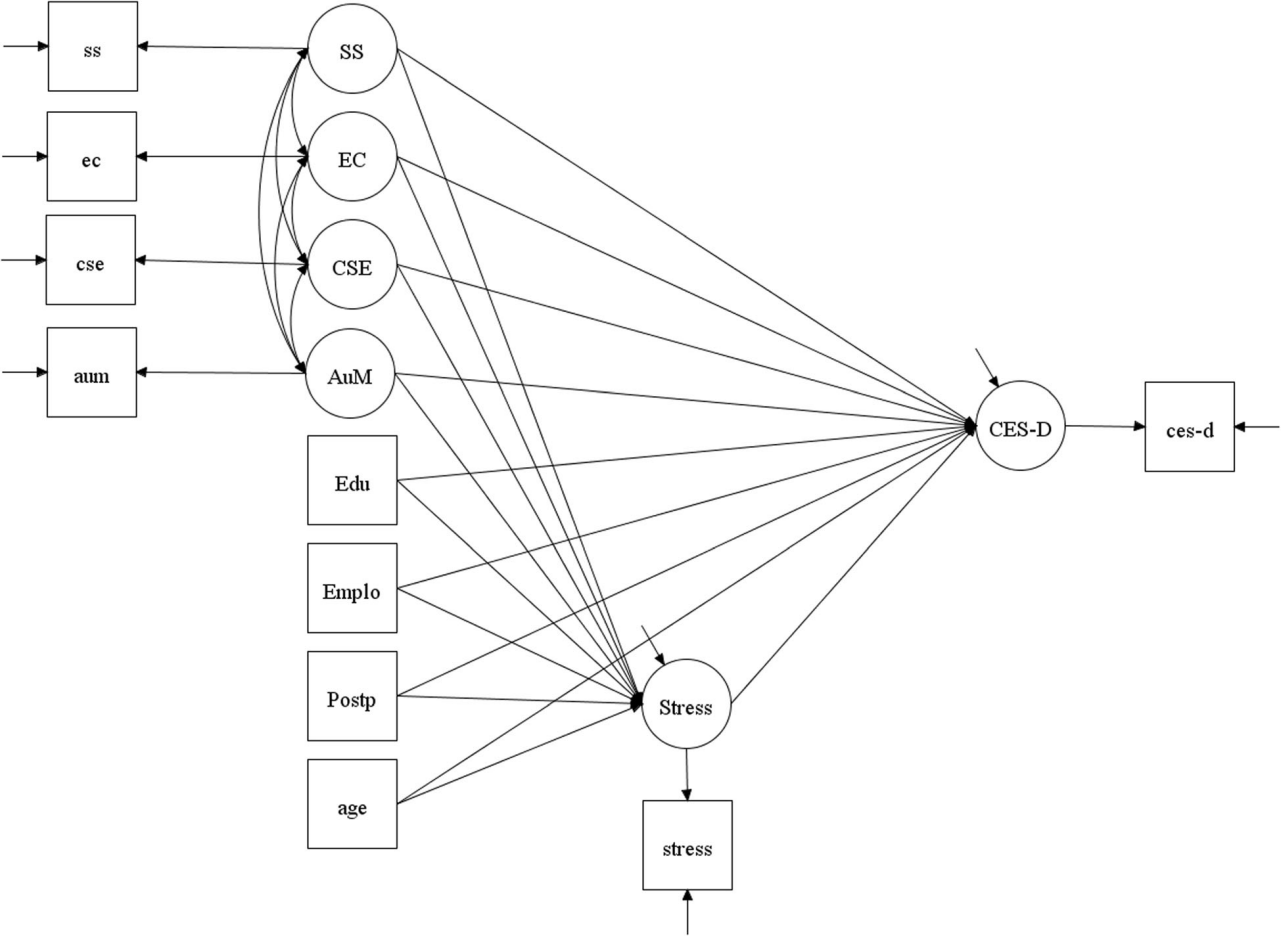
Model Testing. SS = social support, EC = emotional coping, CSE = coping self-efficacy, AuM = autonomous motivation, Edu = education, Emplo = employment status, Postp = postpartum status, CES-D = depressive symptoms. The exogenous variables (independent variables or predictors) were social support, emotional coping, coping self-efficacy, and autonomous motivation. Covariates were education, employment status, postpartum period, and age

Mplus version 8 [40] was used for all analyses. The exogenous variables (independent variables or predictors) were social support, emotional coping, coping self-efficacy, and autonomous motivation. The endogenous variable (outcome or dependent variable) was depressive symptoms. The mediator in the model was perceived stress. Covariates were education, employment status, postpartum period, and age. Education was dichotomized as high school or less education vs. at least some college education. Employment status was coded as a dummy variable: employed vs. unemployed. Postpartum period and age were continuous variables. Figure 1 presents the study framework for mediation model testing. Composite indicator structural equation (CISE) modeling using maximum likelihood estimation was performed to examine whether perceived stress helped to account for any associations between social support, emotional coping, coping self-efficacy, autonomous motivation, and depressive symptoms. The CISE is an errors-in-variables approach that models measurement errors in both exogenous and endogenous variables. The CISE creates latent variables by combining the items of each separate measurement domain into a single indicator [41]. To control for measurement errors in the CISE, the error variance of each indicator was fixed at (1–α)*σ^2^ (where α = Cronbach’s alpha, and σ^2^ = the observed variance of the composite variable) [42]. Proportion of maximum possible (POMP) scores in the endogenous variable per unit change in the exogenous variable was used to compute effect sizes: POMP = [parameter estimate/[(maximum scale value–minimum scale value)+ 1)]*100 [43]. POMP ranges from 0 to 100% and can have a positive or negative value depending on the direction of the parameter estimate. Regardless of the direction, the larger the value, the larger the effect size. Model fit was assessed using a R^2^ value for the endogenous variable.

## Results

### Demographics

Of 1544 women screened, 956 women met the study criteria. The key reason for not being qualified was body mass index outside ranges of 25.0–39.9 [7]. Of 956 qualified women, 740 completed the baseline phone interview. The key reason for not completing the interview was being unable to reach the participants, such as the phone being disconnected. Table 1 presents demographics of the study sample (*N* = 740). The mean age was 28.06 years (SD = 5.12) and mean postpartum period was 1.67 years (SD = 1.26). Most women were non-Hispanic White (74.6%) and non-smokers (71.5%), had at least some college education (64.1%), and were obese (63.4% BMI 30–39.9 kg/m^2^). About 43% of the women were employed full- or part-time.

**Table 1:** Demographics of the Study Participants: Low-Income Overweight or Obese Women (*N* = 740)

Variable	Number (*%*) or mean (*SD*)
Age (years)	28.06 (5.12)
Postpartum period (years)	1.67 (1.26)
Race	
Non-Hispanic Black	188 (25.4)
Non-Hispanic White	552 (74.6)
Smoking status	
Never smoked	328 (44.3)
Smoked, but quit	201 (27.2)
Current smoker	211 (28.5)
Education	
High school or less	266 (30.0)
Some college or technical school	338 (45.7)
College graduate or higher	136 (18.4)
Employment status	
Full-time	165 (22.3)
Part-time	154 (20.8)
Unemployed	160 (21.6)
Homemaker	180 (24.3)
Self-employed	23 (3.1)
Student	44 (5.6)
Other	14 (1.9)
Body Mass Index category	
Overweight (25.0–29.9 Kg/m^2^)	271 (36.6)
Obese category 1 (30.0–34.9 Kg/m^2^)	269 (36.4)
Obese category 2 (35.0–39.9 Kg/m^2^)	200 (27.1)

*N* = 740

### Total effects

As shown in Table 2 (total effects), there were significant and negative overall associations between social support and depressive symptoms (b [unstandardized parameter estimate] = − 3.21, *p* < 0.01, POMP = − 5.83%) and between coping self-efficacy and depressive symptoms (b = − 7.69, *p* < 0.01, POMP = − 13.99%). However, there were not significant overall associations between emotional coping and depressive symptoms or between autonomous motivation and depressive symptoms. The R^2^ value was 0.66 for depressive symptoms, reflecting that the mediation model explained 66% of the variance in depressive symptoms.

**Table 2:** Direct and Indirect Paths for Low-Income Overweight or Obese Mothers of Young Children

	b (standard error)	95% Confidence interval	***P***-value	β	POMP %
**Total Effects**					
SS➔ DEP	−3.21 (0.58)	−4.36, −2.07	< 0.01	−0.24	−5.83
EC ➔ DEP	−1.36 (1.64)	− 4.53, 1.91	0.41	− 0.06	− 2.47
CSE ➔ DEP	−7.69 (0.96)	−9.61, − 5.90	< 0.01	− 0.42	−13.99
AUM ➔ DEP	0.29 (0.30)	− 0.32, 0.84	0.33	0.04	0.53
**Direct Effects**					
SS ➔ STR	−0.11 (0.03)	−0.16, − 0.06	< 0.01	−0.19	−3.88
EC ➔ STR	−0.20 (0.08)	−0.34, − 0.05	0.01	−0.20	−7.09
CSE ➔ STR	−0.39 (0.04)	−0.48, − 0.31	< 0.01	−0.51	−13.99
AUM ➔ STR	−0.00 (0.02)	−0.03, 0.02	0.81	−0.01	− 0.10
STR ➔ DEP	19.37 (1.90)	15.72, 22.89	< 0.01	0.81	35.21
**Indirect Effects (Mediation)**					
SS ➔ STR➔ DEP	−2.10 (0.54)	−3.15, −1.11	< 0.01	−0.16	−3.81
EC ➔ STR➔ DEP	−3.81 (1.61)	−7.13, −0.97	0.02	−0.16	−6.92
CSE ➔ STR➔ DEP	−7.53 (1.15)	−9.88, −5.53	< 0.01	−0.41	−13.69
AUM ➔ STR➔ DEP	−0.07 (0.29)	−0.67, 0.47	0.82	−0.01	− 0.12
**Direct Effects**					
SS ➔ DEP	−1.11 (0.57)	−2.25, −0.05	0.05	−0.08	−2.02
EC ➔ DEP	2.45 (1.73)	−0.79, 5.72	0.16	0.10	4.45
CSE ➔ DEP	−0.17 (1.18)	−2.40, 2.19	0.89	−0.01	−0.31
AUM ➔ DEP	0.36 (0.29)	−0.24, 0.91	0.21	0.05	0.66

*b* unstandardized parameter estimate, *β* standardized parameter estimate, *POMP* Proportion of maximum possible (POMP) scores in the endogenous variable per unit change in the exogenous variable (outcome variable = depressive symptoms), *SS* Social support, *EC* Emotional coping, *CSE* Coping self-efficacy, *AUM* Autonomous motivation, *STR* Perceived stress, *DEP* Depressive symptoms. The exogenous variables (independent variables or predictors) were social support, emotional coping, coping self-efficacy, and autonomous motivation. Covariates were education, employment status, postpartum period, and age

### Direct and indirect (mediation) effects

**Fig. 2:**
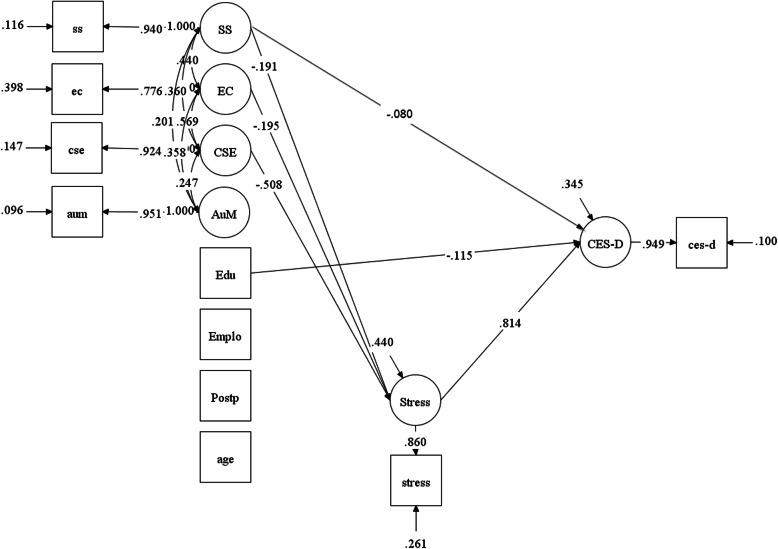
Significant Paths of Model Testing. This significant path is defined as *p* < 0.05. SS = social support, EC = emotional coping, CSE = coping self-efficacy, AuM = autonomous motivation, Edu = education, Emplo = employment status, Postp = postpartum status, CES-D = depressive symptoms. The exogenous variables (independent variables or predictors) were social support, emotional coping, coping self-efficacy, and autonomous motivation. Covariates were education, employment status, postpartum period, and age

Figure 2 shows significant paths of the mediation model. There were significant and negative associations between social support and perceived stress (b = − 0.11, *p* < 0.01, POMP = − 3.88%), between emotional coping and perceived stress (b = − 0.20, *p* < 0.05, POMP = − 7.09%), and between coping self-efficacy and perceived stress (b = − 0.39, *p* < 0.01, POMP = − 13.99%). Yet, there was not a significant association between autonomous motivation and perceived stress. When controlling for social support, emotional coping, coping self-efficacy, and autonomous motivation, perceived stress was significantly and positively associated with depressive symptoms (b = 19.37, *p* < 0.01, POMP = 35.21%). When investigating the potential role of perceived stress as a mediator, the indirect effects of social support on depressive symptoms (b = − 2.10, *p* < 0.01, POMP = − 3.81%), emotional coping on depressive symptoms (b = − 3.81, *p* < 0.05, POMP = − 6.92%), and coping self-efficacy on depressive symptoms (b = − 7.53, *p* < 0.01, POMP = − 13.69%) through perceived stress were significant and negative. This means that perceived stress might help explain the associations between social support, emotional coping, coping self-efficacy, and depressive symptoms. Yet, the indirect effect of autonomous motivation on depressive symptoms through perceived stress was not significant. When controlling for perceived stress, only social support was directly associated with depressive symptoms (b = − 1.11, *p* = 0.05, POMP = − 2.02%), but emotional coping, coping self-efficacy, and autonomous motivation were not significantly associated with depressive symptoms.

#### Covariates

Education was negatively associated with depressive symptoms (b = − 1.23, *p* < 0.01, POMP = − 2.23%) but was not associated with perceived stress. Employment status, postpartum status, and age were not significantly associated with depressive symptoms or perceived stress.

## Discussion

The present study findings represent potentially important contributions to understanding the role and potential consequences of perceived stress, thus potentially informing future stress management interventions aimed at alleviating depressive symptoms. This is because this study explores the role of perceived stress as a potential mediator that helps explain the associations between the psychosocial factors (social support, emotional coping, coping self-efficacy, and autonomous motivation) and depressive symptoms in low-income overweight or obese mothers of young children. Results of the present study partially supported the study hypotheses: perceived stress served as a potential mediator in the associations between social support, emotional coping, coping self-efficacy, and depressive symptoms.

The present study showed that increased social support, emotional coping, and coping self-efficacy were significantly associated with lower levels of perceived stress, all of which support postulates from the Self-Determination Theory [44]. Social support is widely recognized as one of the most important buffers to the stress response [44]. Despite the use of different stress measurements (e.g., economic stress and stress in general) across studies, the present and prior studies consistently showed that increased social support was associated with decreased stress [12, 13, 23, 24]. Literature has documented that quality of coping is associated with short-and long-term outcomes of exposure to stressful life events [44]. Emotional coping, which can be used when individuals have no control over the situation, has sometimes been mislabeled as a maladaptive approach because prior emotional coping measurements have been confounded with distress and self-deprecation [45]. A recent study showed that emotional coping was an adaptive approach [45], which is consistent with the present study demonstrating that increased emotional coping was associated with lower levels of perceived stress. The potential helpfulness of emotion coping in reducing stress may be related to low-income women living in stressful daily life situations many of which are out of women’s control. Coping self-efficacy can also influence perceived stress and one’s ability to deal with perceived stress [44]. The result of the current study shows increased coping self-efficacy is associated with decreased perceived stress, which is in line with prior studies of mothers of young children [11, 29–31].

The present study failed to detect an association between autonomous motivation and perceived stress, which does not support one of the postulates of the Self-Determination Theory. There are a number of possible explanations. First, participants’ levels of autonomous motivation were measured by asking them to report the extent to which particular personal values or interests related to health motivated them to engage in activities to manage stress. It is possible that the values or interests in the questions did not relate well to the values and interests of the research participants. Thus, even if they had high levels of autonomous motivation, perhaps the measure did not effectively identify them as having such motivation. Also, individuals with high autonomous motivation tend to perceive life events as challenging instead of stressful [44]. Therefore, even if a person with a high level of autonomous motivation identified times when the situations in the perceived stress scale were encountered, their acknowledgement of that might not have actually reflected perceived stress in their minds (but, rather, challenge). To successfully manage stress, high-autonomous-motivation individuals will need to cope adaptively [44]. This might suggest an interaction between autonomous motivation and emotional coping that was not included in our analyses. A characteristic of autonomous motivation is interest-taking, which encourages openness, exploration, and growth, thereby building knowledge and skills and providing a foundation for development. When individuals take a self-interest in an outcome, they are more likely to effectively regulate negative emotion in response to stressful situations [44]. Relations with autonomous motivation assume that individuals with high autonomous motivation can implement adaptive approaches to successfully manage stress. Such assumptions might not be as applicable to our priority population or the situations they face, as these mothers face many constraints and stressors (for example, economic stress, violence exposure [46]) that are outside of their control. Thus, it is possible that such uncontrollable stressors prevent them from implementing successful strategies to reduce stress even if they have high levels of autonomous motivation.

Results of the study revealed that participants with higher levels of perceived stress are likely to report more depressive symptoms, which supports the findings of prior studies of low-income mothers of young children [10–14]. The present study also shows a relatively large magnitude of effect (35.2%) for the association between perceived stress and depressive symptoms, meaning that a 1-point decrease on the 4-point perceived stress scale is associated with 35% reduction of depressive symptoms. The prevalence of depression in U.S. adults has increased significantly from 2005 (about 6.6%) to 2015 (about 7.3%), especially for the low-income group [47]. Low-income mothers of young children are less likely to receive any form of treatment for depression even though they experience more severe depressive symptoms than higher-income mothers of young children [1]. Stress has significant and negative impacts on mental health, such as depression, and contributes to mental health disparities [48]. Therefore, the present study findings are consistent with the potential importance of conducting stress management interventions to alleviate depressive symptoms, which could also help potentially reduce health disparities.

Our findings showed that perceived stress could plausibly mediate the association between social support and depressive symptoms, the association between emotional coping and depressive symptoms, and the association between coping self-efficacy and depressive symptoms. The case of emotional coping is interesting. Lack of a “total effect” of emotional coping on depressive symptoms alongside an indirect effect through stress suggests that some aspect of emotional coping might also be associated with greater depressive symptoms or with some other variable(s) that help to produce depressive symptoms. The patterns for social support and coping self-efficacy were more typical in that they each showed “total effect” relations with depressive symptoms that were markedly reduced when the potential mediator of stress was included in the model (dropping to non-significance in the case of coping self-efficacy). In contrast to social support, emotional coping, and coping self-efficacy, however, perceived stress did not serve as a plausible mediator for any association between autonomous motivation and depressive symptoms (also no overall relation between autonomous motivation and depressive symptoms was found).

Consistent with these findings, future stress management intervention studies aimed at alleviating depressive symptoms in low-income overweight or obese mothers of young children might consider including practical strategies to increase social support, emotional coping, and coping self-efficacy. A potential strategy to increase social support might include positive ways to solicit social support. Given the constraints that might limit influences of autonomous motivation, potential strategies to promote positive emotional coping might be crucial. Such strategies might include intervention content aimed at reducing negative thinking, such as self-blame. If mothers can begin to reappraise problematic challenges as lessons learned and opportunities to do better the next time, stress or depression might be reduced. Other strategies could include undertaking enjoyable activities to manage emotions or reframing situations more positively [49], and increasing relaxed awareness of current emotional states [44]. A potential strategy to boost coping self-efficacy might include an emphasis on taking small steps and applying simple and practical strategies to identify the root cause of perceived stress (for example considering what, why, when, where, who, and how to react) then problem solving accordingly.

After controlling for the other psychosocial variables and for perceived stress, the study showed a significant negative association between social support and depressive symptoms, suggesting increased social support decreased depressive symptoms. This finding is consistent with prior studies of low-income mothers of young children [12, 27]. However, the study failed to observe either total effects or direct effects of emotional coping on depressive symptoms when controlling for the other psychosocial variables and perceived stress. The overall lack of relation is consistent with a prior study of postpartum women [28]. The current study did not observe the association between coping self-efficacy and depressive symptoms when controlling for stress. We also failed to detect an association between autonomous motivation and depressive symptoms. Prior depression intervention studies of middle-aged adults have evaluated such association and have yielded mixed results [50, 51]. Future studies are needed to evaluate the association between autonomous motivation and depressive symptoms.

There are certainly limitations to the study. First, this study utilized cross-sectional data to test the mediation effects, which does not warrant causal conclusions [52]. However, evidence has shown the importance of cross-sectional mediation analyses to provide valuable insight into potential mechanisms, especially when there are strong theoretical foundations to substantiate the hypothesized influences [53, 54]. Our mediation model was tested based on a strong theoretical foundation (the Self-Determination Theory) to direct the modeled influences. Moreover, the exogenous measures in the model (i.e., social support, emotional coping, coping self-efficacy, and autonomous motivation) were assessed retrospectively, which provides a time sequence that would allow for influences over time that parallel the structure of the model.

Another potential limitation is that the study used a self-reported perceived stress measure instead of an objective measurement such as hair cortisol. Yet, hair cortisol is not associated with stress in low-income mothers of young children [55]. This study only measured perception of stress in general but not specific types of stress, such as work stress or parenting stress. These different types of stress might differentially contribute to depressive symptoms. In addition, depressive symptoms were self-reported instead of representing a diagnosis of depression from an electronic health record, which is not feasible or practical for researchers collecting data in community settings. Our sample only included Non-Hispanic Black and White participants because our collaborating sites served a very small number of women with other racial/ethnic backgrounds. Finally, the sample was predominately Non-Hispanic White, which was similar to the racial/ethnic backgrounds of WIC enrollees at our collaborating sites but not WIC nationwide in the U.S. Thus, the study findings might not apply to higher income women or other low-income women who are minorities, such as Hispanic or Asian. However, the study findings contribute and advance scientific and mental health research in low-income overweight or obese mothers of young children.

## Conclusion

Depression adversely affects health outcomes of low-income mothers of young children and their children. In a large sample of low-income overweight or obese mothers of young children, the study findings identified perceived stress as a potentially helpful explanation for relations of social support, emotional coping, and self-efficacy with depressive symptoms. Such findings would be consistent with use of stress management interventions in attempts to alleviate depressive symptoms in the priority population. However, the study did not show a relation between autonomous motivation and perceived stress or depressive symptoms. Future stress management intervention studies aiming to alleviate depressive symptoms in the priority population might consider including practical strategies to promote social support, emotional coping, and coping self-efficacy to reduce stress, which might potentially decrease depressive symptoms.

## Data Availability

The datasets generated and/or analyzed during the current study are not publicly available because we are in the stage of data analysis to answer other research questions but are available from the corresponding author on reasonable request.

## Abbreviations

BMI: Body mass index
CISE: Composite indicator structural equation
α: Cronbach’s alpha
POMP: Proportion of maximum possible
U.S.: United States
WIC: The special supplemental nutrition program for women, infants, and children
